# HelexKids 2.0: A linguistically annotated lexical database building on HelexKids

**DOI:** 10.3758/s13428-026-03179-7

**Published:** 2026-10-07

**Authors:** Anthi Revithiadou, Aris Terzopoulos, Georgia Niolaki, Giorgos Markopoulos, Konstantinos Avdelidis, Ilias Mittas, Kosmas Kosmidis

**Affiliations:** 1 School of Philology, Department of Linguistics, Aristotle University of Thessaloniki, University, Campus 54124, Thessaloniki, Greece; 2 Department of Psychology, Birmingham City University, Birmingham, UK; 3 School of Education, University of Birmingham, Birmingham, UK; 4 Department of Mediterranean Studies and School of Philology, Department of Linguistics, University of the Aegean and University of Ioannina, Rhodes & Ioannina, Greece; 5 School of Physics, Aristotle University of Thessaloniki, Thessaloniki, Greece

**Keywords:** Lexical database, Greek, Elementary school vocabulary, Morphophonological annotation, Educational resources

## Abstract

HelexKids 2.0 represents an annotated expansion of the original HelexKids, a word frequency database compiled from Greek and Cypriot elementary school textbooks. This article presents the extended annotation methodology employed to enrich the database with part-of-speech tags, phonological properties (syllable count, phonetic transcription, orthographic and phonological syllabification, and stress), and morphological features (case, number, gender, and inflection class for nouns). All original frequency and lexical measures are retained, and new ones (e.g., PLD20, bigram and biphone frequencies) are added. The annotation process combined automated tools with extensive manual annotation and expert verification. We redesigned the database to assemble dynamically from the full set of textbooks, delivering results on the fly. The annotated database comprises 67,802 types and over 1.3 million tokens. We report on stress pattern distributions across major parts of speech and grade levels, show how stress is distributed by word class, and provide hierarchies of word-level syllabic templates. HelexKids 2.0 is freely available through the GRADIENCE project webpage and serves as a valuable resource for psycholinguistic and morphophonological research, as well as for educational applications.

**Supplementary Information:**

The online version contains supplementary material available at https://doi.org/10.3758/s13428-026-03179-7.

## Introduction

Word frequency databases have become valuable resources for psycholinguistic research, educational assessment, and computational linguistics. They provide researchers with reliable data on word distributions and frequencies, assist educators in selecting vocabulary appropriate for different age groups, and offer linguists insights into both systematic and exceptional patterns in language. While numerous frequency databases exist for adults across several languages (Brysbaert & New, 2009; Keuleers et al., 2010), child-specific databases are relatively rare. This gap is significant because lexical exposure differs substantially between adults and children, a difference that proves important when selecting appropriate vocabulary items for teaching or when designing research materials (Beckman & Edwards, 2000; Massaro, 2015; Montag & MacDonald, 2015; Storkel, 2004).

For Greek, *HelexKids* (Terzopoulos et al., 2017; https://www.helexkids.org) was developed as the first psycholinguistic database specifically for primary school children. Based on official textbooks used across six grade levels in primary schools in Greece and Cyprus, HelexKids offers frequency information that reflects the vocabulary children aged 6–12 are exposed to in the classroom. The database reveals several important characteristics of children’s reading materials (Terzopoulos et al., 2017). First, approximately 50% of words at each grade level were hapax legomena, that is, words appearing only once in the total textbooks. Second, words were shorter and more frequent compared to adult Greek databases (e.g., *SUBTLEX-GR*, Dimitropoulou et al., 2010; *GreekLex 2*, Kyparissiadis et al., 2017). Finally, the top 100 words accounted for 44.9% of all tokens.

However, although the original *HelexKids* provided frequency and basic distributional metrics, it did not include grammatical information. For many research applications, particularly in phonological development, morphophonological analysis, literacy acquisition, and the design of intervention materials for speech and writing difficulties, more detailed linguistic annotation is required (Terzopoulos et al., 2020). Greek, in particular, presents a range of challenges that underscore the need for fully annotated lexical databases, especially those targeting child learners. The language has morphology-dependent stress (see Alderete, 1999, 2001; Idsardi, 1992; Kiparsky, 1973, 1984; Kiparsky & Halle, 1977; Melvold, 1990; Revithiadou, 1999, among others), which entails that stress is not predictable; it can surface on any of the final three syllables (see Apoussidou, 2007; Burzio & Tantalou, 2007; Drachman & Malikouti-Drachman, 1999; Malikouti-Drachman & Drachman, 1989; Ralli & Touratzidis, 1991; Revithiadou, 1999, among others). Furthermore, the nominal system exhibits multiple inflectional classes (Anastassiadis-Symeonidis, 2012; Ralli, 2000, 2005) with distinct phonological and morphological properties that interact with stress placement (Apostolouda, 2018; Revithiadou & Lengeris, 2016; Revithiadou et al., 2026a, b; Sims, 2019).

Morphophonological properties also differ systematically between verbs and nominals. For instance, verb stress is generally more predictable and is determined by morphological features such as tense (past tense forms are typically proparoxytone) or by conjugation class properties (non-past 1st person singular forms with a theme element display final stress, whereas those without a theme element are paroxytone). Verbs and nouns also differ in their phonotactic patterns: for example, the cluster /vɣ/, consisting of a voiced labio-dental and a dorsal fricative occurs word-initially in verbs (e.g., [ˈvɣazo] ‘remove/take out’), whereas the sequence /vð/ (e.g., [vðoˈmaða] ‘week’), consisting of a voiced labio-dental and a coronal fricative, occurs in nouns.

Quantifying the presence of such systematic patterns, as well as the extent of idiosyncratic properties, is crucial, especially for the design of experimental materials and for the development of instructional and reading resources for young speakers (Korochkina et al., 2024). The annotated version of HelexKids, named *HelexKids* *2.0*, addresses exactly this gap for Greek. More specifically, it adopts a richer linguistic annotation scheme incorporating part-of-speech (PoS) tags, morphological features for nominal inflection, and phonological properties including detailed stress pattern information, phonetic (IPA) transcription, orthographic and phonological syllabification, and word-level syllable templates. In this respect, the annotated HelexKids can also serve as an example for other languages, since it offers a broad range of annotation parameters.

This article has three main objectives. First, we present the annotation methodology employed in HelexKids 2.0, including a detailed description of the semi-automated pipeline that combined existing resources, machine learning approaches, and principled manual annotation and verification. Second, we describe the database content, including information on the distribution of major word classes as well as the distribution of phonological characteristics (stress, word length, word-level syllable structure), per grade level or grade range. Third, we demonstrate the contribution of HelexKids 2.0 to theoretical research on the morphophonological structure of Greek, as well as to literacy instruction and pedagogical practice.

## Database annotation

In this section, we present the steps undertaken for the morphophonological annotation of HelexKids. The original lexical database comprises 76 textbooks and workbooks (some spanning multiple volumes) covering Greek language, arts, mathematics, science, history, and other subjects taught in elementary education (Grades 1–6) in Greece and Cyprus. The majority of these books are used exclusively at a single grade, with 16 shared across two or more grades.^1^ The textbooks were developed starting in 2007, officially approved in 2013, and are still in use. These books provide the 67,802 annotated word entries (types) of HelexKids 2.0 across six grades*,* and over 1.3 million tokens.

The database preserves the grade-level organization of the original HelexKids, with separate lexicons for each grade level (1–6) and cumulative grade ranges (Grades 1–2, 1–3, 1–4, 1–5, and 1–6). In addition, HelexKids 2.0 can generate lexicons for other grade combinations (e.g., Grades 2–3, 2–4, 3–5). To reflect the actual material a student encounters, for books shared across grades, type and token counts in cumulative grades are assigned to the grade where each book is first introduced and are not recounted in later grades in which the same book appears (see section “The HelexKids 2.0 database: Descriptive statistics”).

Each entry includes all annotations described in sections “PoS annotation”, “Development of PoS tag list”, “Phonological annotation”, “Morphological feature annotation”: PoS category, syllable count, IPA transcription, orthographic and phonological syllabification, word-level syllable templates, stress pattern, and morphological features (nouns only).

In addition to the morphophonological annotations, HelexKids 2.0 retains all variables from the original database, including raw frequency counts, estimated frequencies per million words (u), Zipf scale values (van Heuven et al., 2014), Orthographic Levenshtein Distance 20 (OLD20; Yarkoni et al., 2008),^2^ Standard Frequency Index (SFI) scores, dispersion measures (D), Contextual Diversity (CD) scores (Adelman et al., 2006), and word length in letters. Phonological Levenshtein Distance 20 (PLD20; Suárez et al., 2011; Yap & Balota, 2009) and bigram and biphone frequency (type and token; Vitevitch & Luce, 1999) were also added in the updated database.

HelexKids 2.0 differs from the original HelexKids in one crucial respect: it has been radically redesigned to be dynamically assembled on demand. Lexical items are extracted from the full set of textbooks, stored morphophonological information is retrieved, and additional metrics (e.g., SFI, D, CD) are computed on the fly (see section “The HelexKids 2.0 database: Structure and interface” for details on the tool’s architecture).

### Pre-processing: Correction of character encoding

Before annotation could proceed, we needed to systematically correct the HelexKids Excel files. During the original compilation process, English letters had been accidentally substituted for visually similar Greek characters (e.g., Latin ‘A’ for Greek ‘Α’, Latin ‘o’ for Greek ‘ο’). This problem stemmed from converting scanned PDF files to Excel format, which introduced character-recognition errors. We systematically identified and corrected all instances to ensure the annotation tools in subsequent stages properly recognized Greek words. A master Excel file containing all words in HelexKids was also prepared, which formed the basis for the annotation process.

### PoS annotation

PoS annotation proceeded through multiple stages, combining existing resources with machine learning approaches, followed by extensive manual annotation and verification. Initial annotation used two existing annotated databases: GreekLex 2 (Kyparissiadis et al., 2017), which covers 35,282 types, and *A-Clean* (Apostolouda, 2018), a manually annotated database with PoS and morphosyntactic features that includes 72,103 types. It was extracted from the *Clean Corpus* (Protopapas et al., 2012), which in turn was derived from a previous version of the *Hellenic National Corpus* (Institute for Language and Speech Processing – Athena Research Center, 2015; https://hnc.ilsp.gr). Automatic matching of these resources using exact word-form comparison reduced the number of untagged items. After this initial matching phase, approximately 45,000 words remained unannotated, representing about two-thirds of the total types in HelexKids 2.0.

Next, we compiled a file of non-annotated lexical items and processed it using spaCy’s PoS tagging models for Greek (Honnibal et al., 2020; version 3.2.4; https://spacy.io/models/el). We tested both the large and the small models to optimize accuracy. However, spaCy significantly underperformed on this task. For instance, certain words received incorrect tags: *τσάντα* [ˈʦanda] ‘bag’ was incorrectly tagged as a proper noun rather than a common noun, and *απελπισία* [apelpiˈsia] ‘despair’ was incorrectly tagged as a verb rather than a noun.

Another challenge concerned forms that are morphosyntactically ambiguous between categories. For example, words ending in -*menos* (-*μενος*) are traditionally categorized as participles, yet many such forms function clearly as adjectives (e.g., *δυστυχισμένος* [ðistiçiˈzmenos] ‘unhappy’) or even nouns (e.g., *εργαζόμενος* [erɣaˈzomenos] ‘employed/employee’) rather than as genuine participles (e.g., *κουρασμένος* [kuɾaˈzmenos] ‘tired’). One criterion for identifying genuine participles is whether a passive perfective construction can be formed, a diagnostic that requires human judgment to apply.

Additionally, since the original HelexKids lacked context, words with syncretism created ambiguity. For instance, the form *διορθώσεις* [ðjorˈθosis] can be either a noun ‘correction’ (plural) or a verb ‘you correct’ (2nd person singular perfective). Moreover, several Greek words function across multiple grammatical categories depending on context: *ένα* [ˈena] can serve as a numeral (‘one’), an indefinite article (‘a/an’), or a pronoun (‘someone/something’). Finally, certain lexical items, such as *μπα* [ˈba] (‘nah, no way, I doubt it’) and *μακάρι* [maˈkaɾi] (‘if only, I wish’), cannot be easily assigned to a specific grammatical category and therefore require human judgment.

For these reasons, we opted to manually annotate the entire list of lexical items based on specific, linguistically motivated criteria established by our research team. These criteria and the methodology applied are discussed in the following section.

### Development of PoS tag list

Because the lexical items in HelexKids lack linguistic context, we developed a comprehensive PoS annotation guide to address those lexical items that were ambiguous with regard to their grammatical category. For this purpose, we relied on authoritative Greek grammatical references including Triantafyllidis (2018 [1941]), Holton et al. (2012), the *Dictionary of Standard Modern Greek* (Institute of Modern Greek Studies, 1998), and the *Practical Dictionary of Modern Greek* (Charalambakis, 2023). The annotation scheme we developed uses compound tags (e.g., ADV/NOUN, NOUN/PNOUN, PCP/ADJ/NOUN) for the lexical items that belong to multiple categories. Table 1 presents representative examples of the simple and compound morphosyntactic categories employed in the annotation.

**Table 1:** Representative categories of PoS annotation

Word	PoS	IPA transcription	Gloss
***βιβλίο***	NOUN	[vi.ˈvli.o.]	‘book’
***παράγοντας***	NOUN/GER	[pa.ˈɾa.ɣo.^n^das.]	‘factor/producing’
***ευθεία***	ADV/NOUN	[ef.ˈθi.a.]	‘straight/line’
***Ειρήνη***	NOUN/PNOUN	[i.ˈɾi.ni.]	‘peace/Irene’
***εργαζόμενη***	PCP/ADJ/NOUN	[er.ɣa.ˈzo.me.ni.]	‘employed/employee’
***εκφράσεις***	VERB/NOUN	[ek.ˈfra.sis.]	‘(you-sg) express/expressions’

After the initial automated tagging attempts, members of the research team manually annotated and corrected the data. We decided that, when genuine ambiguity existed (i.e., when a word could legitimately function in multiple grammatical categories and no context was available to disambiguate), compound tags were required to capture this multifunctionality.

To be certain that the annotation scheme was applied consistently and reliably, we initially implemented a quality control process for PoS annotation consistency. Three researchers independently annotated a random sample of 1000 words. Inter-rater reliability was assessed using Krippendorff’s *α* (Krippendorff, 2004), which yielded *α* = 0.789. This value falls below the threshold for reliable data (α ≥ 0.800) but exceeds the minimum for tentative conclusions (α ≥ 0.667). Disagreements were resolved through discussion and consultation with the team’s linguists and authoritative reference works. Most disagreements pertained to whether all applicable PoS tags had been included, rather than to misclassification of PoS category itself.^3^

As a result, annotation guidelines highlighting points of difficulty were developed and shared with the annotators. The annotation scheme contained 61 distinct PoS categories and category combinations. (See Supplementary Information, Appendix B, Table B1 for all categories in HelexKids 2.0.) The corrected annotations were then reinserted into the master Excel file, completing the PoS annotation of HelexKids 2.0.

### Phonological annotation

Words in HelexKids 2.0 were annotated for five phonological properties: number of syllables, orthographic and phonological syllabification, stress pattern, and word-level syllable templates. Custom R scripts were developed to automate these annotations (R Core Team, 2024; version 4.4.0). All phonological annotations required manual checking to ensure accuracy.

#### Orthographic and phonological syllabification

Orthographic syllabification was performed using a custom R script designed to follow Greek syllabification conventions (Petrounias, 2002).^4^ The script implements standard rules for consonant cluster division, treating digraphs (e.g., *μπ, ντ, γκ*) as units and applying the *Maximum Onset Principle* (Kahn, 1976; Selkirk, 1982), whereby consonants are assigned to the onset of the following syllable whenever permitted by the phonotactic constraints of the language.

However, the orthographic syllabification applied to HelexKids 2.0 differs from traditional syllable division rules in several crucial respects. More specifically, it can treat vowel sequences such as *ια*, *ιά*, *εια*, *ειά*, *ιο*, *ιό*, etc. as one syllable, depending on the lexical entry. Phonemically, these sequences are realized either as a glide or palatal consonant followed by a vowel within a single syllable, or as two independent vowels split across separate syllables (see, e.g., Apostolopoulou, 2018; Baltazani et al., 2016; Baltazani & Topintzi, 2012; Markopoulos & Apostolopoulou, 2020; Rytting, 2005; Topintzi & Baltazani, 2016). Their exact syllabification depends on the particular lexical item and the learned/non-learned vocabulary distinction (Anastassiadis-Symeonidis & Fliatouras, 2004, 2018; Fliatouras & Anastassiadis-Symeonidis, 2019; Rytting, 2005). In examples like *μά.τια* [ˈma.tça.] ‘eyes’ and *α.λή.θεια* [a.ˈli.θça.] ‘truth’, the orthographic *ι* and *ει* correspond to a palatal consonant. However, in *διαφωνείτε* [ðja.fo.ˈni.te.] ‘(you-pl) disagree’ the letter *ι* corresponds phonetically to a glide, whereas in *διαγράφω* [ði.a.ˈɣra.fo.] ~ [ðja.ˈɣra.fo.] ‘I delete’, there is variation between a full vowel and a glide (see also the discussion in section “Syllable count and stress annotation”). By contrast, traditional orthographic syllabification rules treat such vowel sequences as heterosyllabic, e.g., *μά.τι.α*, *α.λή.θει.α*.^5^ Similarly, letter combinations such as *γγ*, which correspond phonemically to a single consonant [ɡ] (e.g., *α.γγα.ρεί.α* [a.ɡa.ˈɾi.a.] ‘drudgery, chore’), are treated as single phonemic entities. Traditional orthographic conventions split such double letters into two syllables, e.g., *αγ.γα.ρεί.α*, resulting in an orthographically closed syllable *αγ* [aɣ.].

IPA transcription and phonological syllabification were carried out concurrently, since both processes are essential for the accurate identification of syllable boundaries and stress placement. Both processes employed a custom R script to generate phonetic representations following standard transcription conventions for Modern Greek (Arvaniti, 1999; Revithiadou, 2021). The script also implemented phonological rules including voicing assimilation, glide strengthening, vowel deletion, etc., and applied phonological syllabification based on the sonority sequencing principle (Kappa, 2002).

Table 2 shows examples of words that are orthographically syllabified, transcribed into IPA, and phonologically syllabified.

**Table 2:** Phonological annotation: Orthographic and phonological syllabification

Word	Orthographic syllabification	IPA syllabification	Gloss
***αγαπητός***	α.γα.πη.τός	[a.ɣa.pi.ˈtos.]	‘lovable’
***μαρμάρινος***	μαρ.μά.ρι.νος	[mar.ˈma.ɾi.nos.]	‘of marble’
***αλήθεια***	α.λή.θεια	[a.ˈli.θça.]	‘truth’

*‘*ˈ’ indicates stress on the following syllable, ‘.’ indicates syllable boundary

#### Word-level syllable templates

Word-level syllable templates were generated using a custom R script that translated IPA symbols and syllable breaks into syllabic templates. We used an established notation system in which V represents a vowel, C represents a consonant, diphthongs are marked as VJ or JV depending on their position (where J represents a glide), and, in coda position, L and N designate liquids and nasals, respectively.

Syllabified word templates provide an abstract representation of syllable structure at the word level that can be used for phonotactic analysis or stimulus selection in psycholinguistic experiments. Table 3 illustrates the syllable templates for representative words.

**Table 3:** Phonological annotation: Word-level syllable templates

Word	CV template	Gloss
***αγαπητός***	V.CV.CV.ˈCVC.	‘lovable’
***μαρμάρινος***	CVL.ˈCV.CV.CVC.	‘of marble’
***αλήθεια***	V.ˈCV.CCV.	‘truth’
***αηδόνι***	VJ.ˈCV.CV.	‘nightingale’

#### Syllable count and stress annotation

Syllable count and stress annotation required both orthographic syllabification and IPA transcription as prerequisites. Using an R script, syllables were counted, and stress position was identified by locating the primary stress marker ‘ˈ’ in phonemic transcription. Thus, words were classified as U (ultimate stress, on the final syllable), PU (penultimate stress, on the second-to-last syllable), APU (antepenultimate stress, on the third-to-last syllable), double-stressed (APU and U), or unstressed.

Several issues emerged during stress annotation that required special handling. First, monosyllabic content words such as *γη* [ˈʝi] ‘earth’ and *ζω* [ˈzo] ‘live’ bear phonological stress but lack orthographic accent marks in Greek orthography according to standard spelling conventions. As a result, automated tools misidentified them as stressless. Second, words written entirely in capital letters (e.g., *ΑΓΑΛΜΑ* [ˈaɣalma] ‘statue’) lack accent marks and were therefore incorrectly categorized as stressless. Third, words bearing clitic stress, such as *άλογό* [ˈaloˈɣo] ‘horse’, carry double accent marks and were initially misclassified as U-stressed. In fact, the U-stress mark here represents clitic stress, while the primary lexical stress is APU. Such items are indicated as “Double Stress” in the database.

For monosyllabic words, the script verified PoS category and assigned stress to content words while marking function words as unstressed. Capitalized words were compared manually with their lowercase versions to recover stress information. In cases of capitalization-induced ambiguity (e.g., *ΑΛΛΑ* [ˈala] ‘other (plural neuter)﻿’ vs. [aˈla] ‘but’), stress was assigned according to the stress pattern of the more frequent lowercase form, as established by frequency counts in the database.

A further complication in syllable counting and stress assignment arose from variation in the phonetic rendering of certain Greek words. The same word may have different pronunciations due to free variation or speaker-dependent factors. Variation occurs particularly in hiatus environments involving sequences such as *ια, ιά, ιο, ιό, ιού*, etc., where vowel sequences can be realized either as separate syllables or as diphthongs or palatal plus vowel sequences (see Discussion in section “Orthographic and phonological syllabification” and references therein). This variation affects syllable count, syllabification patterns, and stress classification. For instance, a word that is APU-stressed in a four-syllable pronunciation becomes PU-stressed in a three-syllable pronunciation; e.g., *α.κρί.βεια* [a.ˈkri.vʝa.] ‘precision, high cost’ has three syllables and is PU-stressed, whereas *α.κρί.βει.α* [a.ˈkri.vi.a.] has four syllables and is APU-stressed. We annotated such words for both stress patterns. The team determined the reported transcriptions for these forms through discussion and consensus. Approximately 3.2% of word types received dual annotations for pronunciation variation, predominantly involving words with vowel sequences in hiatus positions.

Table 4 presents the syllable count and stress patterns for representative examples, shown also in orthographic and phonological syllabification.

**Table 4:** Phonological annotation: Syllable count, syllabification, and stress pattern

Word	Syllables (*N*)	Orthographic syllabification	IPA syllabification	Stress
***αγαπητός***	4	α.γα.πη.τός	[a.ɣa.pi.ˈtos.]	U
***μαρμάρινος***	4	μαρ.μά.ρι.νος	[mar.ˈma.ɾi.nos.]	APU
***αλήθεια***	3	α.λή.θεια	[a.ˈli.θça.]	PU
***άλογό***	3	ά.λο.γό	[ˈa.lo.ˈɣo.]	Double Stress
***το***	1	το.	[to.]	Unstressed
***ακρίβεια***	3/4	α.κρί.βεια/α.κρί.βει.α	[a.ˈkri.vʝa.] [a.ˈkri.vi.a.]	PU/APU
***αντρειοσύνη***	4/5	α.ντρειο.σύ.νη/α.ντρει.ο.σύ.νη	[a.ⁿdrjo.ˈsi.ni.] [a.ⁿdri.o.ˈsi.ni.]	PU

APU: Antepenultimate stress, PU: Penultimate stress, U: Ultimate stress

*Glosses*. Lovable, of marble, truth, horse, the (singular neuter), precision/high cost, courage/bravery

### Morphological feature annotation

Morphological feature annotation in HelexKids 2.0 focused on noun inflection to extract stress frequency patterns across different noun classes. Because nouns exhibit greater stress-pattern complexity than other word classes, and stress distributions differ across inflection classes (see Discussion in section “Introduction”), we annotated nouns in greater detail. Specifically, we manually annotated nouns for inflectional endings and morphological features, including case, number, and gender. A random sample of 1000 words was independently annotated by three researchers, and an inter-annotator agreement was assessed using Krippendorff’s *α* (*α* = 0.827), indicating strong agreement. The team’s linguists resolved disagreements through discussion. Most disagreements arose from the omission of some possible morphosyntactic features that a word type could be associated with. After this process, we established a set of guidelines highlighting the errors most likely to occur and shared them with the annotators. Table 5 presents representative examples of the morphological annotation of nouns.

**Table 5:** Representative examples of morphological annotation for nouns

Word	PoS	Ending	Morphological features	IPA syllabification	Gloss
***άβακα***	NOUN	asM	SgGenAcc	[ˈa.va.ka.]	‘abacus’
***αβγά***	NOUN	oN	PlNomAcc	[a.ˈvɣa.]	‘eggs’
***αβιταμίνωση***	NOUN	iF	SgNomAcc	[a.vi.ta.ˈmi.no.si.]	‘vitamin deficiency’
***άγαλμα***	NOUN	maN	SgNomAcc	[ˈa.ɣal.ma.]	‘statue’

Sg: Singular, Pl: Plural; Gen: Genitive, Acc: Accusative, Nom: Nominative; M: Masculine, N: Neuter, F: Feminine

## The HelexKids 2.0 database: Structure and interface

In this section, we outline the dynamic redesign of the original HelexKids, which was carried out to make the database more functional and extensible.^6^

### Database architecture and storage format

HelexKids 2.0 is implemented as a relational database in MariaDB (version 10.11.19; MariaDB Foundation, 2026). It consists of nine interconnected tables organized in a modular architecture that separates linguistic annotation, text- and grade-level metadata, and quantitative measures, allowing users to pose queries across various linguistic dimensions. The central *helexkids_words* table contains 67,802 types (representing over 1.3 million tokens) with comprehensive linguistic annotation. The architecture supports both word-level and database-level analyses through interconnected tables storing pre-computed frequency metrics (including raw frequency, frequency per million, dispersion, and contextual diversity) and book-specific word occurrences. Metadata tables organize the database hierarchically by book type, curriculum unit, textbook instruction period, and grade level, while stored query templates enable efficient retrieval of word lists based on these parameters.

This relational structure allows researchers to extract word lists filtered by multiple simultaneous criteria (e.g., syllable count, stress pattern, morphological class, frequency thresholds, grade level) and to examine how vocabulary patterns change across different grade levels. These backend design choices formed the basis for the user-facing tools described in the following section.

## Web interface and search capabilities

HelexKids 2.0 is openly accessible to users through a web-based interface hosted on the GRADIENCE project webpage (see fn 4). To accommodate both international and Greek-speaking researchers and educators, the interface is available in English and Greek. It also provides users with direct access to the word frequency database, documentation on updates relative to the original HelexKids, license and citation information, and a bilingual user guide.

The search and filtering interface consists of seven collapsible filter panels that allow users to query the database using multiple criteria simultaneously:

- **Grade(s) Selection:** Users can select either a single grade (1–6) or a cumulative grade range (1–3 or 4–6), with type and token counts updating dynamically.
- **Part of Speech (PoS):** A scrollable multi-select list allows filtering by one or more grammatical categories. Entries with mixed PoS labels (e.g., ADJ/NOUN) are returned whenever any selected category matches a component of their annotation.
- **Surface Form Search:** Users can retrieve words by internal letter sequence (Word Contains) or orthographic ending (Word Ends With).
- **Syllables and Stress:** A range slider controls syllable count. Stress patterns can be filtered via checkboxes (e.g., APU, PU, U, unstressed); a separate dropdown enables filtering by the CV structure of the stressed syllable (e.g., ˈCV, ˈCVC).
- **Frequency and** **Distribution:** Range sliders control seven standard metrics: Zipf value, frequency per million, log frequency per million, dispersion, contextual diversity, estimated frequency per million, and Standard Frequency Index.
- **Lexical Neighborhood:** Two range sliders implement Levenshtein-based neighborhood density filtering: OLD20 and PLD20.
- **Bigram & Biphone** **Frequencies**: Four range sliders operating on a logarithmic scale provide filtering by bigram and biphone frequency by type and token count.

Search results are displayed in a sortable table format showing word forms, frequency counts, PoS tags, phonological properties, and (for nouns) morphological features. Any column can sort results to facilitate identification of high-frequency items, rare words, or items with specific phonological properties. The interface supports exporting results in CSV or Excel format with UTF-8 BOM encoding, which ensures proper handling of Greek and IPA characters across different software applications and operating systems.

Results are retrieved from pre-computed frequency metrics stored in the backend to minimize response times and are dynamically filtered according to the criteria specified by the user. After updates or corrections to the database, the cache files are refreshed to keep the metrics up to date.

## The HelexKids 2.0 database: Descriptive statistics

In this section, we provide an overview of the descriptive statistics of HelexKids 2.0, including the distribution of PoS and stress patterns, as well as syllable-related measures such as syllable count, average syllable length, and frequent word-level syllable templates. We conducted all analyses in R (R Core Team, 2024, version 4.4.0).

We begin by examining how lexical coverage develops across the six grade levels represented in the database. Table 6 shows the distribution of the 67,802 word types and also reports token counts as they appear per grade, without deduplication of books shared across grades (see section “Database annotation”); that is, the tokens of a book shared by two or more grades are counted again in each grade in which it appears. We observe that types and tokens increase at every grade, but not at a constant rate. The largest expansion is between Grades 2 and 3 (+87.98% types, +139.91% tokens), due to the increase in the number of school subjects introduced in Grade 3. From this point on, the growth rate slows steadily, reaching its lowest point between Grades 5 and 6 (+7.05% types, +14.94% tokens).

**Table 6:** Word types and tokens per grade (non-cumulative) with grade-to-grade differences (Δ) and relative growth rates (%)

Grade	Types (*N*)	Δ Types (*N*)	Δ Types (%)	Tokens (*N)*	Δ Tokens	Δ Tokens (%)
**1**	9077	__	__	70,345	__	__
**2**	11,575	2498	27.52	95,886	25,541	36.31
**3**	21,759	10,184	87.98	230,041	134,155	139.91
**4**	33,322	11,563	53.14	338,202	108,161	47.02
**5**	44,383	11,061	33.19	463,502	125,300	37.05
**6**	47,511	3128	7.05	532,749	69,247	14.94

While Table 6 presents how much material appears at each grade, Table 7 illustrates how much total material a student has encountered by that point in schooling by providing cumulative type and token counts across grade ranges. This perspective allows us to trace how vocabulary accumulates over time. As shown in Table 7, token counts increase steadily with grade level due to the accumulation of text material. However, the degree of increase is not uniform. The smallest raw increase in types occurs early, between Grade 1 and Grades 1–2 (4530 new types), with every subsequent step adding more. Notably, by Grade 6, where the whole vocabulary is available, the growth rate relative to Grades 1–5 shrinks considerably: approximately 14% for types and 24% for tokens relative to the previous grade step. This pattern occurs because shared books within grade ranges are counted only at the grade at which they are first introduced. Still, growth is considerable from Grades 1–3 through 1–5. For example, in Grades 1–3, types increase by 49% and tokens by nearly 63% compared to Grades 1–2.

**Table 7:** Type and token counts across grade ranges (cumulative) with grade-to-grade differences (Δ) and relative growth rates (%)

Grade Range	Types (*N*)	Δ Types	Δ Types (%)	Tokens (*N*)	Δ Tokens	Δ Tokens (%)
**1**	9077	__	__	70,345	—	__
**1–2**	13,607	4530	33.3	136,880	66,535	48.62
**1–3**	26,691	13,084	49	366,921	230,041	62.7
**1–4**	40,922	14,231	34.77	637,854	270,933	42.48
**1–5**	58,374	17,452	29.89	1,051,783	413,929	39.35
**1–6**	67,802	9428	13.9	1,379,521	327,738	23.76

### PoS distribution

The three most frequent categories are nouns (329,644 tokens, 23.90%), verbs (211,743 tokens, 15.35%), and function words such as pronouns and articles (PRN/ART: 185,380 tokens, 13.44%), which form a compound category due to their shared forms (syncretism). This distribution reflects the discourse characteristics of educational texts, which tend to be noun-heavy and make frequent use of the definite article, a key feature of Greek grammar.

Table 8 presents the frequency distribution of major PoS categories. Content words dominated the lexicon in type counts: nouns (21,606), verbs (20,575), and adjectives (10,452) collectively accounted for nearly 78% of all types. However, these three categories represented just under 44% of total word tokens (nouns: 23.90%; verbs: 15.35%; adjectives: 4.68%). Function words showed the opposite pattern, with minimal vocabulary diversity but high token frequency. For example, articles comprised only three types but accounted for 52,102 tokens (3.78%). Similarly, the combined pronoun/article category (PRN/ART) represented just 11 types, yet accounted for 185,380 tokens (13.44% of the database). Prepositions (48 types, 52,435 tokens) and conjunctions (56 types, 80,747 tokens) displayed comparable patterns. (See Supplementary Information, Appendix B, Table B1 for a complete PoS breakdown.)

**Table 8:** Distribution of PoS categories across types and tokens in HelexKids 2.0

PoS	Types (*N*)	Types (%)	Tokens (*N*)	Tokens (%)
**NOUN**	21,606	31.87	329,644	23.90
**VERB**	20,575	30.35	211,743	15.35
**PRN/ART**	11	0.02	185,380	13.44
**CNJ**	56	0.08	80,747	5.85
**ADJ**	10,452	15.42	64,543	4.68
**PREP**	48	0.07	52,435	3.80
**ART**	3	0.00	52,102	3.78

The stark type–token asymmetry between content and function words reflects a well-established distributional pattern in natural language: nouns, verbs, and adjectives account for most lexical diversity, whereas a small set of high-frequency function words accounts for a large share of total tokens. These differences between types and tokens became more apparent through PoS annotation, transforming the database from a simple word list into a resource for examining how the grammatical patterns of Greek school texts evolve across grades.

### Stress pattern distribution

#### Stress patterns in major parts of speech

**Fig. 1:**
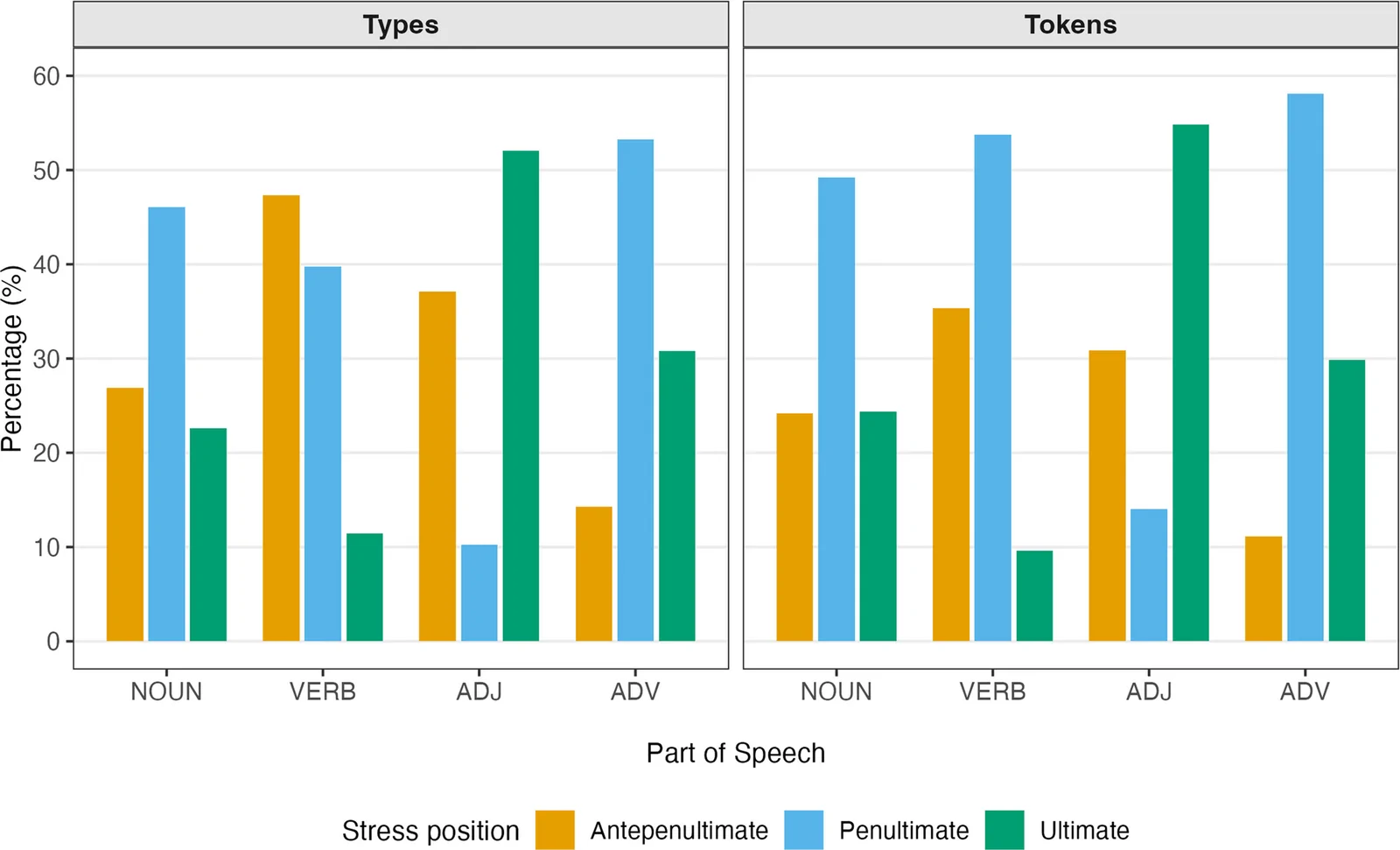
Distribution of stress by major PoS in HelexKids 2.0. The *left panel* shows the percentage of types and the *right panel* shows the percentage of tokens. *Bars* represent the percentage of words with APU (*orange*), PU (*blue*), and U (*green*) stress for nouns, verbs, adjectives, and adverbs

As a morphology-dependent system with a three-syllable window restriction, Greek permits stress on the APU, PU, and U syllable. Across all morphosyntactic words in HelexKids 2.0, stress pattern distributions vary systematically. Figure 1 presents the distribution of APU, PU, and U stress in verbs, nouns, adjectives, and adverbs, calculated over types and tokens. (Detailed percentage distributions are provided in Supplementary Information, Appendix B, Tables B2–B3.)

Nouns exhibit relatively stable distributions across type and token frequencies. They favor PU stress (types: 46.03%; tokens: 49.23%), with APU and U stress occurring at moderate frequencies. Verbs show the highest proportion of APU stress in both types (47.35%) and tokens (35.31%) compared to other morphosyntactic categories. However, types show a closer competition between APU (47.35%) and PU (39.76%) stress than tokens do (PU: 53.75% vs. APU: 35.31%).

Adjectives show a dramatically different pattern: final stress is the predominant stress pattern in both types (52.08%) and tokens (54.81%), while PU stress is relatively low (types: 10.26%; tokens: 14.01%). On the other hand, adverbs show the strongest preference for PU stress (types: 53.22%; tokens: 58.10%); U stress occurs at elevated frequencies compared to other categories (types: 30.81%; tokens: 29.87%), while APU stress occurs at rather low frequencies (types: 14.29%; tokens: 11.10%).

Such cross-category differences indicate that word class plays a significant role in Greek stress patterns, with each major word category showing distinct distributional properties. This, in turn, underscores the importance of HelexKids 2.0 as a large, developmentally stratified database that enables the systematic investigation of phonology–morphology interactions across word categories and frequency profiles.

#### Stress patterns in nouns and verbs across grade ranges

The grade-level organization of HelexKids 2.0 allows examination of how children’s exposure to different stress patterns changes across elementary education. Researchers can investigate stress at individual grade levels (e.g., vocabulary specific to Grade 3 or Grade 5) or at cumulative grade ranges (e.g., all vocabulary encountered through Grade 3 or through Grade 5).

**Fig. 2:**
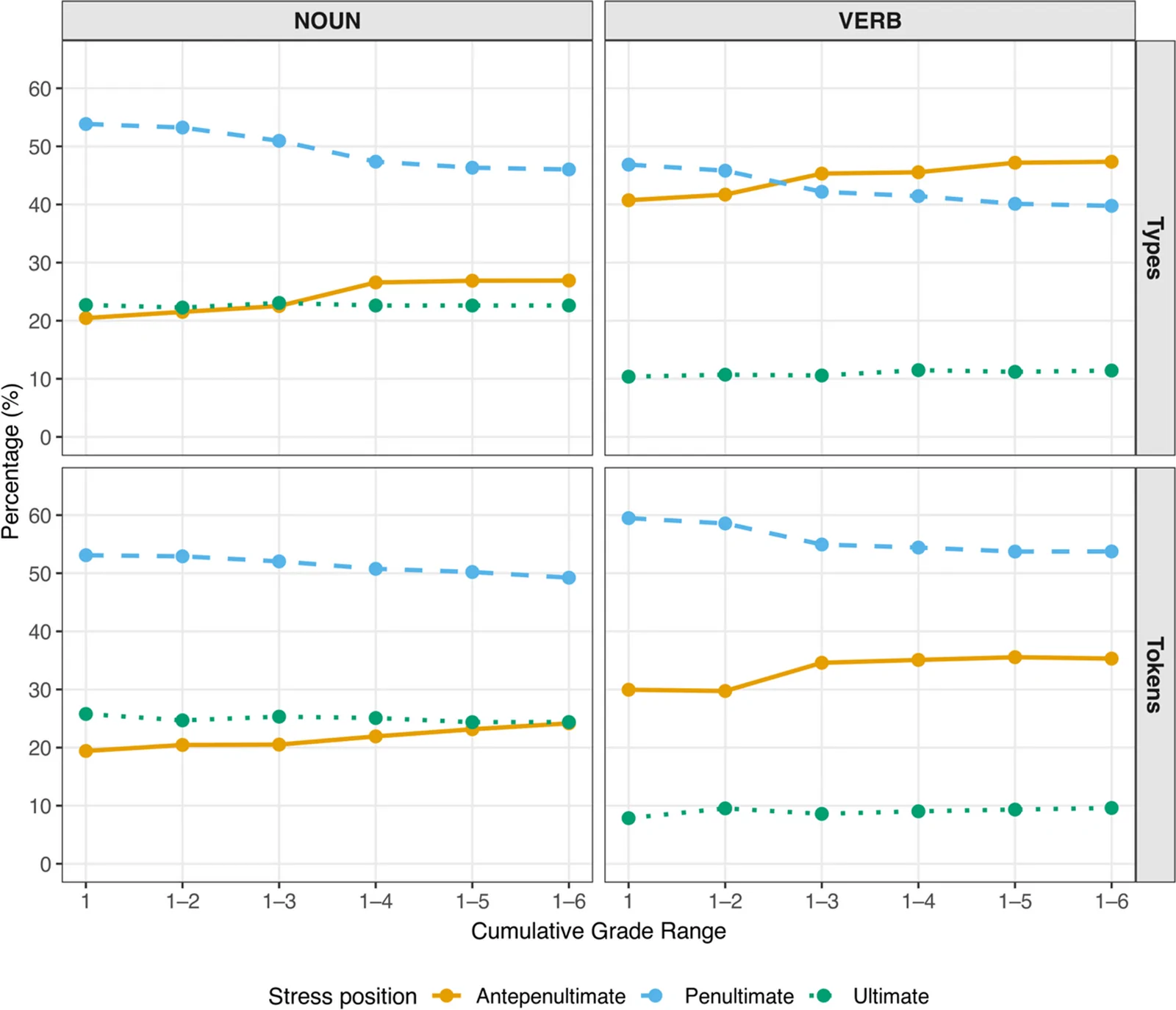
Cumulative distribution of stress patterns in nouns and verbs across grade ranges (Grade 1 through Grades 1–6). The *top row* displays percentages of type counts; the *bottom row* displays percentages of token counts. Nouns are shown in the *left panels* and verbs in the *right panels*. *Lines* are color-coded by stress position: APU (*solid orange*), PU (*dashed blue*), and U (*dotted green*)

Figure 2 illustrates how stress patterns are distributed across the noun and verb vocabulary that accumulates from Grade 1 through Grade 6. It offers a picture of the specific grammatical categories and their associated stress patterns that students have been exposed to by the end of a given grade. A cumulative perspective better reflects children’s vocabulary exposure across schooling. Tracking how stress is distributed within and across grammatical categories, and how this distribution shifts as cumulative vocabulary changes, may also reveal when certain stress patterns become more or less prominent in children’s input. (Detailed percentage breakdowns are provided in Supplementary Information, Appendix B, Tables B4–B7.)

The data reveal distinct patterns for nouns versus verbs that remain relatively stable as vocabulary accumulates across grade levels. For nouns, PU stress consistently prevails across all grade ranges, accounting for 46–54% of types (top left, Fig. 2) and 49–53% of tokens (bottom left, Fig. 2). APU and U stress show broadly comparable proportions in the type analysis, though they diverge slightly: APU ranges from approximately 20–27%, whereas U remains remarkably stable at around 23% throughout. In the token-based analysis, U stress is more prominent than APU at Grade 1 (25.78% vs. 19.41%), but the gap closes gradually across grade levels (24.39% vs. 24.17% by Grades 1–6). Notably, both the type and token distributions show a gradual shift across grades. More specifically, from Grade 1 to Grades 1–6, APU stress increases from 20.46% to 26.91% in types and from 19.41% to 24.17% in tokens, while PU stress decreases correspondingly from 53.85% to 46.03% in types and from 53.10% to 49.23% in tokens. U stress remains virtually unchanged across all grade ranges (22.71% to 22.62% in types; 25.78% to 24.39% in tokens). Since the rise in APU (~6–7 percentage points in types, ~5 in tokens) closely mirrors the decline in PU (~8 percentage points in types, ~4 in tokens), the gradual shift in the noun distribution reflects a redistribution specifically between these two stress patterns as the noun vocabulary expands, with U stress not benefiting from this change.

Verbs display a notably different distribution and a more pronounced type-token divergence. In the type analysis, APU and PU stress occur at broadly comparable frequencies, with their relative prominence reversing across grades: APU increases from 40.73% at Grade 1 to 47.35% at Grades 1–6, while PU decreases from 46.86% to 39.76% (top right, Fig. 2). The token-based distribution tells a different story: PU-stressed verbs are substantially more frequent in the textbooks (54–59% of tokens), while APU accounts for only 30–36% of tokens (bottom right, Fig. 2). This gap – around 12 percentage points between APU types (47.35%) and APU tokens (35.31%) at Grades 1–6, and around 14 percentage points between PU types (39.76%) and PU tokens (53.75%) – indicates that high-frequency verbal forms in children’s textbooks are predominantly PU-stressed, despite APU verbal forms being more numerous in the cumulative vocabulary by the later grades. U stress remains low in verbs, representing 10–11% of types and 8–10% of tokens.

Taken together, the systematic type-token divergence observed for verbs, but not nouns, reveals a fundamental difference between the two categories: the most frequent verbal forms in children’s textbooks are predominantly PU-stressed, whereas for nouns, type and token distributions do not show substantial differences.

### Syllable count, average syllable length and most frequent word-level syllable templates across grades

**Fig. 3:**
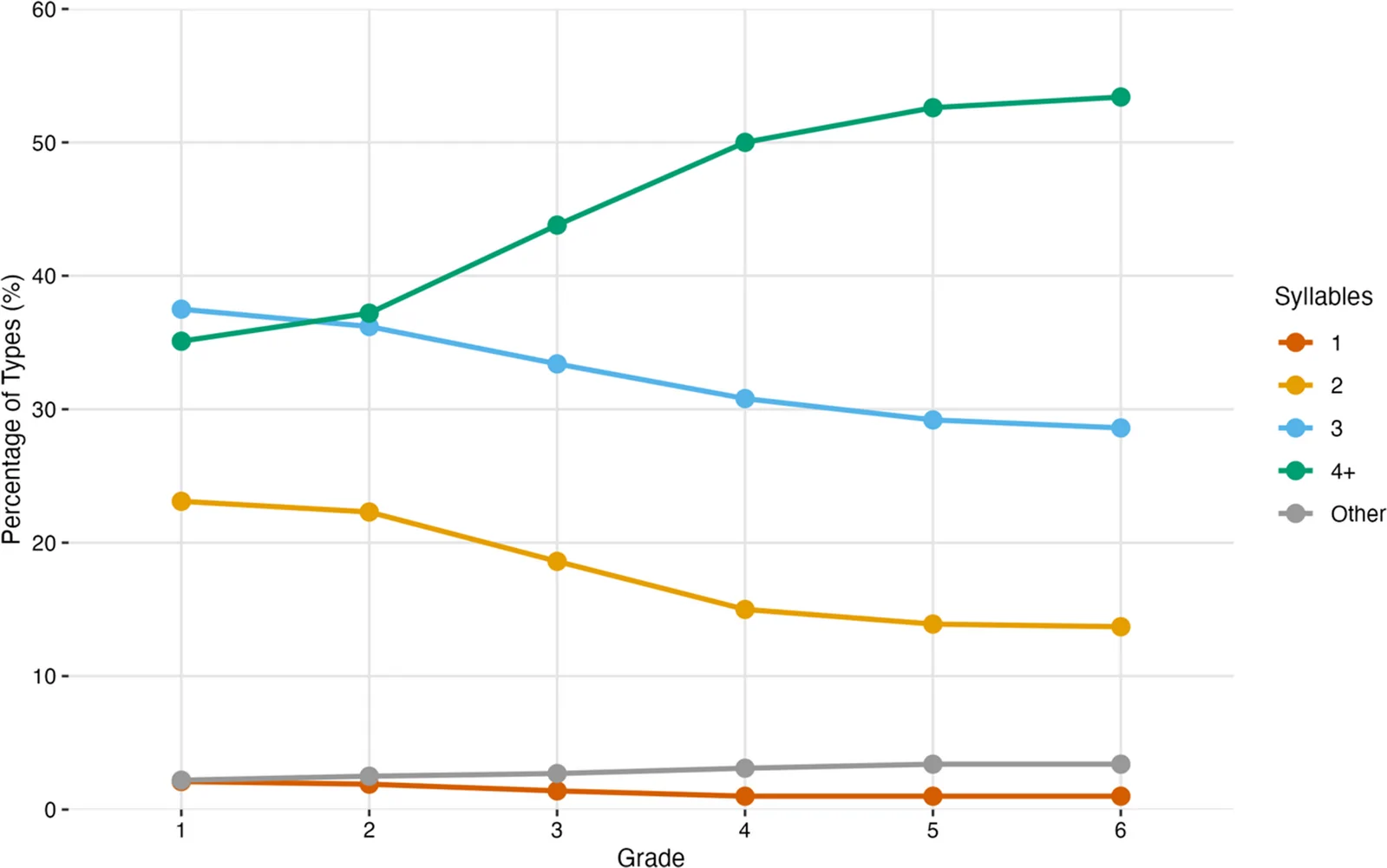
Percentage of word types by syllable length across Grades 1–6. Words with 4+ syllables increase steadily across grades, while two- and three-syllable words decline. One-syllable words remain consistently slightly above zero. Words with variable syllable counts (e.g., 2/3), under “Other”, remain low but increase slightly by Grade 6

Analysis of HelexKids 2.0 reveals clear grade-level differences in syllable count distribution, average syllable length, and the prevalence of specific syllable templates at the word level. Figure 3 displays the percentage of one-syllable, two-syllable, three-syllable, and four-syllable words (types) across grade levels. As the grade level increases, the proportion of disyllabic words decreases (from 23.1% in Grade 1 to 13.7% in Grade 6). This shift pertains to the relative distribution of word lengths, not the raw frequency with which shorter words appear. Although the proportion of disyllabic words decreases across grades, their raw number increases as the overall database grows, rising from 2,101 words in Grade 1 (out of 9077 total words) to 6491 in Grade 6 (out of 47,511 total words). Thus, shorter words do not decrease in absolute terms; instead, they account for a smaller share of a vocabulary that expands with longer words. At the same time, longer words (4+ syllables) increase both in proportion (from 35.1% in Grade 1 to 53.4% in Grade 6) and in absolute count (from 3183 to 25,356). Three-syllable words show a decrease across grades (from 37.5% in Grade 1 to 28.6% in Grade 6), even though their absolute count still increases (from 3403 to 13,604). Words with variable syllable counts (e.g., two or three syllables (2/3)) represent a small proportion of the data (2.1–3.4% across grades). In general, at higher grades, children encounter progressively longer words.

Additionally, average syllable length increases across grade levels, from 3.25 syllables in Grade 1 (*SD* = 1.11, *N* = 9077 words) to 3.75 syllables in Grade 6 (*SD* = 1.20, *N* = 47,511 words). Detailed descriptive statistics are reported in Table 9.

**Table 9:** Mean syllable length of types by grade level, with standard deviations, total word counts, and the proportion of ambiguously coded syllables

Grade	Avg length	SD	Grade types (*N*)	Ambiguous syllables (*Ν*)	Ambiguous syllables (%)*
**1**	3.25	1.11	9077	190	2.1
**2**	3.30	1.13	11,575	278	2.4
**3**	3.47	1.14	21,759	587	2.7
**4**	3.66	1.18	33,322	1036	3.1
**5**	3.73	1.20	44,383	1495	3.4
**6**	3.75	1.20	47,511	1597	3.4

*Note. Ambiguous cases (e.g., 2/3 syllables) were coded as the midpoint

Table 10 illustrates that CV.ˈCV.CV. is the most common word template in types, ranking first consistently across all individual grades. It represents PU-stressed trisyllabic words consisting solely of open syllables.

**Table 10:** CV.ˈCV.CV. as the most frequent syllable template across all grade levels: Type counts and percentages

Grade	Template types (*N*)	Template types (%)	Grade types (*N*)
**1**	441	4.86	9077
**2**	481	4.16	11,575
**3**	666	3.06	21,759
**4**	846	2.54	33,322
**5**	1011	2.28	44,383
**6**	1069	2.25	47,511

Percentage reflects the proportion of word types in each grade displaying this template

The second and third most frequent word templates are ˈCV.CV.CV. (1.78–2.91%) and ˈCV.CV. (1.65–3.9%), with APU and PU stress, respectively. Templates with consonant clusters (e.g., ˈCCV.CV.) are less frequent than those with simple CV structure (see Supplementary Information, Appendix B, Table B8 for the top ten templates per grade by type).

Analysis of word-level syllable templates weighted by token frequency per grade (with no deduplication of shared books) showed that monosyllabic templates were most frequent across all grades. The CV. template ranked first in every grade, accounting for approximately 20–23% of word tokens. Together, the monosyllabic structures in the top 10 (CV., V., CVC., CVN., CCV.) accounted for approximately 30–35% of tokens per grade. Among disyllabic templates, ˈCV.CV. was the most frequent (3.38–4.77%), followed by ˈV.CV. (3.35–4.05%) and CV.ˈCV. (2.04–2.93%). The CV.ˈCV.CV. template, which ranked among the most frequent in the type-based analysis, appeared in the token top 10 only for Grades 1 to 4, ranking 7th–10th (1.59–2.47%), and dropped out of the top 10 from Grade 5 onward. (See Supplementary Information, Appendix B, Table B9, for the top ten templates per grade by tokens.)

## Discussion

### Contributions to research on Greek morphophonology

The extended morphophonological annotation in HelexKids 2.0 significantly enhances the resource. For instance, the distributional data on stress patterns across different PoS and inflection classes offer empirical backing for theories of stress assignment in Greek (Malikouti-Drachman & Drachman, 1989; Revithiadou, 1999). Specifically, the predominance of APU stress in specific noun classes (e.g., -*ma*_*N*_), as opposed to the preference for PU stress in others (e.g., -*a*_*F*_), supports accounts that differentiate between morpholexically conditioned stress and the phonologically determined default (see, e.g., Revithiadou et al., 2026b). On the other hand, the association of verb stress predominantly with PU stress, as opposed to the more even spread of stress patterns in nouns, supports proposals that nouns are prosodically more complex than verbs (e.g., Smith, 1997, 1999).

Additionally, the morphophonological annotation of lexical databases opens new possibilities for systematically exploring how phonological and morphological properties of words interact. Researchers can test, for example, predictions about systematicity and productivity by examining stress patterns or other phonological properties (e.g., phonotactics, syllable structure) within specific morphosyntactic categories or structures (see, e.g., Dabouis & Fournier, 2023; Storme, 2024). The results of such investigations can in turn refine or challenge existing accounts of the morphophonological characteristics of words, thus contributing to broader debates on lexical representation and grammatical architecture.

Furthermore, HelexKids 2.0 recognizes the gradient and variable nature of phonological outputs by offering dual annotations for words with variable pronunciations. This approach acknowledges the ongoing debates about phonological variation in hiatus environments within Greek (Apostolopoulou, 2018; Markopoulos & Apostolopoulou, 2020; Rytting, 2005), and serves as a point of reference for similar annotation projects.

### Linguistic research and educational applications

From a linguistic perspective, the grade-level organization of HelexKids 2.0 allows researchers to examine how children’s exposure to phonological and morphological patterns changes across elementary education: morphological diversity increases across grades, with longer words appearing in later grades. Consistent with this pattern, increases in morphological complexity across grade levels have been reported among English-speaking elementary school learners (*N* = 1052): spelling accuracy of morphemes develops sharply as children move from Grade 2 to Grade 3, due to the more structured teaching of morphemes from Grade 3 onward (Niolaki et al., 2025).

For educational purposes, the database reflects the type of vocabulary children encounter in school texts as instruction gradually introduces less frequent vocabulary as well as more morphologically complex structures, such as derived words, whose morphological structure has been shown to facilitate, rather than hinder, word recognition in children (e.g., Quémart et al., 2012). Thus, HelexKids 2.0 can support the development of reading and spelling materials that draw on morphological structure.

The database also offers researchers the opportunity to investigate frequency effects in morphophonological acquisition and processing. Because both type and token frequencies are available for all annotated categories, researchers can use different frequency measures depending on their research question. For instance, they can examine whether high-frequency stress patterns, word categories, or inflection classes are acquired earlier by children (see, e.g., Ambridge et al., 2015; Bybee, 2001, 2010; Tomasello, 2003) or have a processing advantage compared to low-frequency structures (see, e.g., Brysbaert et al., 2018). Distributional input statistics (e.g., bigram/trigram frequencies) could also be used to test their role in identifying morphological sub-word units (Rastle & Davis, 2003).

Pedagogically, word frequency similarly predicts spelling accuracy across primary grades, with its contribution increasing with literacy experience (e.g., Lété et al., 2008), and repeated exposure supports the formation of orthographic representations for novel or unfamiliar words (van Viersen et al., 2022). Teachers can likewise draw on the PoS and morphological feature annotations to select grade-appropriate vocabulary, also guided by frequency information, since children are more likely to effectively learn words they encounter repeatedly in their school readings (Nagy & Anderson, 1984).

The stress pattern annotations are particularly useful for Greek educators, as stress position is a well-documented difficulty for Greek children in both spelling and reading (Protopapas et al., 2013). Teachers can use HelexKids 2.0 to generate word lists with specific stress patterns for targeted stress instruction, starting from high-frequency word groups with less variable stress patterns before moving to less common ones with more unpredictable stress.

### Comparison with other child-specific databases

HelexKids 2.0 joins a growing collection of child-specific word frequency databases across languages, including *MANULEX* for French (Lété et al., 2004), *childLex* for German (Schroeder et al., 2015), *ESCOLEX* for Portuguese (Soares et al., 2014), and *CYP-LEX* for English (Korochkina et al., 2024), among others. These databases vary in their depth of linguistic annotation: ESCOLEX, childLex, and CYP-LEX provide lemmatization, part-of-speech information, and word length. ESCOLEX additionally includes syllable count and syllable structure information but does not provide phonetic transcriptions.

Complementing this body of work, HelexKids 2.0 extends the annotation scope by including phonological information, serving as a basis for similar annotation practices in other languages. Cross-linguistic comparisons of stress pattern distributions in child lexical databases could illuminate universal and language-specific aspects of children’s early vocabulary. Such comparisons have implications for research on how children acquire the phonological and morphological properties of their lexicon. Finally, richly annotated databases are particularly valuable for stimulus selection in experimental designs, as they allow researchers to target or control specific stress patterns (see, for example, Niolaki et al., 2020; Terzopoulos et al., 2020).

### Limitations and future directions

Several limitations of the current version of HelexKids 2.0 should be acknowledged. Morphological annotation currently covers only nominal inflection. Future versions should include morphological annotation of the remaining categories to provide a complete morphological profile of all lexical items. Greek verbal morphology shows interesting interactions between stem allomorphy, tense/aspect marking, and stress position (Ralli, 2005; Spyropoulos, 2023), which calls for more detailed verb annotation in the future.

Furthermore, the database is based exclusively on textbook materials. Because textbook content and vocabulary are shaped by curricular, pedagogical, and practical constraints rather than children’s productive or receptive vocabulary, the database may not fully capture children’s actual linguistic exposure. In addition, the textbooks themselves cover a specific time period (2007–2013), and new textbooks are scheduled for introduction from 2027 onward. As curricula evolve and new digital educational materials have already been approved for use in schools, future versions should consider incorporating more recent materials as well as those from children’s literature. The expandable architecture of HelexKids 2.0 facilitates future updates, and the annotation pipeline described in this article can be readily extended to new materials with minimal modification.

Finally, the current database focuses on word-level properties and does not include information on syntactic contexts or collocations because the source database consisted of isolated lexical items without sentential context. Context-sensitive annotation and word co-occurrence statistics for exploring the relation between lexical and morphosyntactic patterns remain an important goal for future versions of the database, particularly in light of the new textbooks available from 2027 onwards. Future improvements could also include the addition of lemmatization, which would allow users to access lemma-level frequency data and identify inflectional and derivational families. Such information would greatly enhance the analytical potential of the database, allowing researchers to move beyond individual word forms and explore vocabulary structure at a deeper morphological level.

## Conclusion

HelexKids 2.0 is a freely accessible annotated resource covering many linguistic and frequency-based dimensions. The sophisticated annotation methodology, which combines automated approaches with extensive manual verification, offers high-quality data for PoS categories, phonological properties, and, in the current version, morphological features for nouns. The database supports both research and education. It supports research in developmental psycholinguistics, phonology, morphology, and computational modeling of Greek, while also serving as a useful tool for teachers and curriculum developers.

By making HelexKids 2.0 openly accessible and by continually improving and extending it, we hope it will become a valuable resource for supporting empirical research on the Greek language, children’s language development, and evidence-based language instruction.

## Supplementary Information

Below is the link to the electronic supplementary material.

Supplementary file1 (PDF 463 KB)

## Data Availability

The database and all relevant materials are freely accessible at https://gradience.lit.auth.gr/index.php/helexkids-2-0/ and https://gradience.lit.auth.gr/index.php/helexkids_2-0/.

## Footnotes

1 Seven books are used exclusively in Grade 1, three are shared between Grades 1 and 2, and seven are exclusive to Grade 2. Nine books appear only in Grade 3, six are shared between Grades 3 and 4, and one spans Grades 4, 5, and 6. Nine books are exclusive to Grade 4, fourteen to Grade 5, six are shared between Grades 5 and 6, and fourteen are exclusive to Grade 6.

2 In HelexKids 2.0, the Zipf and OLD20 values from the original database have been recalculated due to minor differences in the total number of lexical items between the two databases. OLD20 was computed for each word type as the mean Levenshtein distance to its 20 closest orthographic neighbors across the full lexicon, using accent-sensitive NFC-normalized lowercase strings with punctuation removed and final sigma (*ς*) mapped to sigma (*σ*). The lowercase normalization follows standard practice in OLD20 computation for Greek (Terzopoulos et al., 2017). The final sigma mapping was not morphologically motivated. In Greek orthography, final sigma is simply the word-final form of sigma rather than a distinct letter, and the distinction carries no morpholexical significance. Collapsing the two therefore entails no loss of linguistically meaningful orthographic information. Without this mapping, the final sigma would be treated as a substitution in word-final position, introducing an artificial penalty. As a result, neighborhood composition would reflect a typographic convention rather than genuine lexical similarity.

3 Words that were difficult to categorize (e.g., *Άη* [ˈaj.] ‘saint’), archaic (e.g., *Αδελφότης* [a.ðel.ˈfo.tis.] ‘brotherhood’*, Επαναστάται* [e.pa.na.ˈsta.te.] ‘revolutionaries’) or Ancient Greek words (e.g., *Αριστεύειν* [a.ɾi.ˈste.vin.] ‘to excel’*, **δικαίοις* [ði.ˈce.is.] ‘to/for the righteous’), dialectal forms (e.g., *έφκιασε* [ˈef.ca.se.] ‘s/he made’*, ήβρανε* [ˈi.vra.ne.] ‘they found’), lexicographically unattested or non-existent items and invented words (e.g., *Αβγατηγανιστάν* [av.ɣa.ti.ɣa.ni.ˈstan.]*, **έφκιακ* [ˈef.ci.ak.]), and words containing orthographic or typographic errors (e.g., *εξαακολουθούν* [e.ksa.a.ko.lu.ˈθun.] instead of *εξακολουθούν* [e.ksa.ko.lu.ˈθun.] ‘they continue’, *τηλεγράφηματα* [ti.le.ˈɣra.fi.ma.ta.] instead of *τηλεγραφήματα* [ti.le.ɣra.ˈfi.ma.ta.] ‘telegrams’) were assigned to the category ‘OTHER’.

4 All codes are openly accessible on the GRADIENCE webpage: https://gradience.lit.auth.gr/index.php/helexkids_2-0/

5 We thank an anonymous reviewer for pointing out that these challenges regarding syllable division rules are not addressed even in the most recent pedagogical grammar used in the final two grades of primary school.

6 See Supplmentary Information, Appendix A for information on HelexKids 2.0 integrity and maintenance, as well as the complete database schema, field specifications, and technical implementation details.

## Code availability

The codes used for the development of HelexKids 2.0 are freely accessible at: https://gradience.lit.auth.gr/index.php/helexkids_2-0/.

## List of abbreviations and symbols

Acc: Accusative
ADJ: Adjective
ADV: Adverb
APU: Antepenultimate stress
ART: Article
C: Consonant
CD: Contextual Diversity
CNJ: Conjunction
D: Dispersion
EXCL: Exclamation
Gen: Genitive
GER: Gerund
IPA: International Phonetic Association
J: Glide
L: Liquid consonant
N: Nasal consonant
NFC: Unicode Normalization Form C (Canonical Decomposition, followed by Canonical Composition)
Nom: Nominative
Pl: Plural
PNOUN: Proper noun
PoS: Part of Speech
PREP: Preposition
PRN: Pronoun
PU: Penultimate stress
SD: Standard Deviation
SFI: Standard Frequency Index
Sg: Singular
U: Ultimate stress
u: Estimated frequency per million
V: Vowel
