# Supplementary material for: HelexKids 2.0: A linguistically annotated lexical database building on HelexKids
Source: Behav Res Methods. 2026 Oct 7;58(11):311. doi: 10.3758/s13428-026-03179-7 (PMC13645859; doi:10.3758/s13428-026-03179-7)
Supplement: Supplementary file 1 [file 13428_2026_3179_MOESM1_ESM.pdf]

Appendices

Appendix A

A1. Database integrity and maintenance

To ensure long-term database integrity and usability, we have implemented several safeguards:

- **Version control**: The database is maintained under version control with clear documentation of any corrections or updates. The current version is HelexKids 2.0, 31/12/2025.
- **Error reporting mechanism**: Users can report suspected annotation errors or inconsistencies through the GRADIENCE email address (gradience@lit.auth.gr). Reported issues are reviewed by the research team, and corrections are incorporated into periodic database updates.
- **Archival plan**: The HelexKids 2.0 database is hosted and maintained on the Aristotle University of Thessaloniki (A.U.Th.) website, where it is made publicly available via a stable institutional URL to ensure long-term access and citability.
- **License***:* *HelexKids* 2.0 is released under a Creative Commons Attribution–NonCommercial 4.0 International (CC BY-NC 4.0) license, which permits non-commercial use, distribution, and adaptation of the database, provided that appropriate credit is given to the authors and the source. Users are instructed to cite both the present article and the original *HelexKids* publication (Terzopoulos et al., 2017) when using the database.

A2. The architecture of HelexKids 2.0

The HelexKids 2.0 database architecture is designed to support simple and complex linguistic queries across six grade levels. Figure A1 presents the internal structure of the database, and below we describe each table and its role in the database ecosystem.

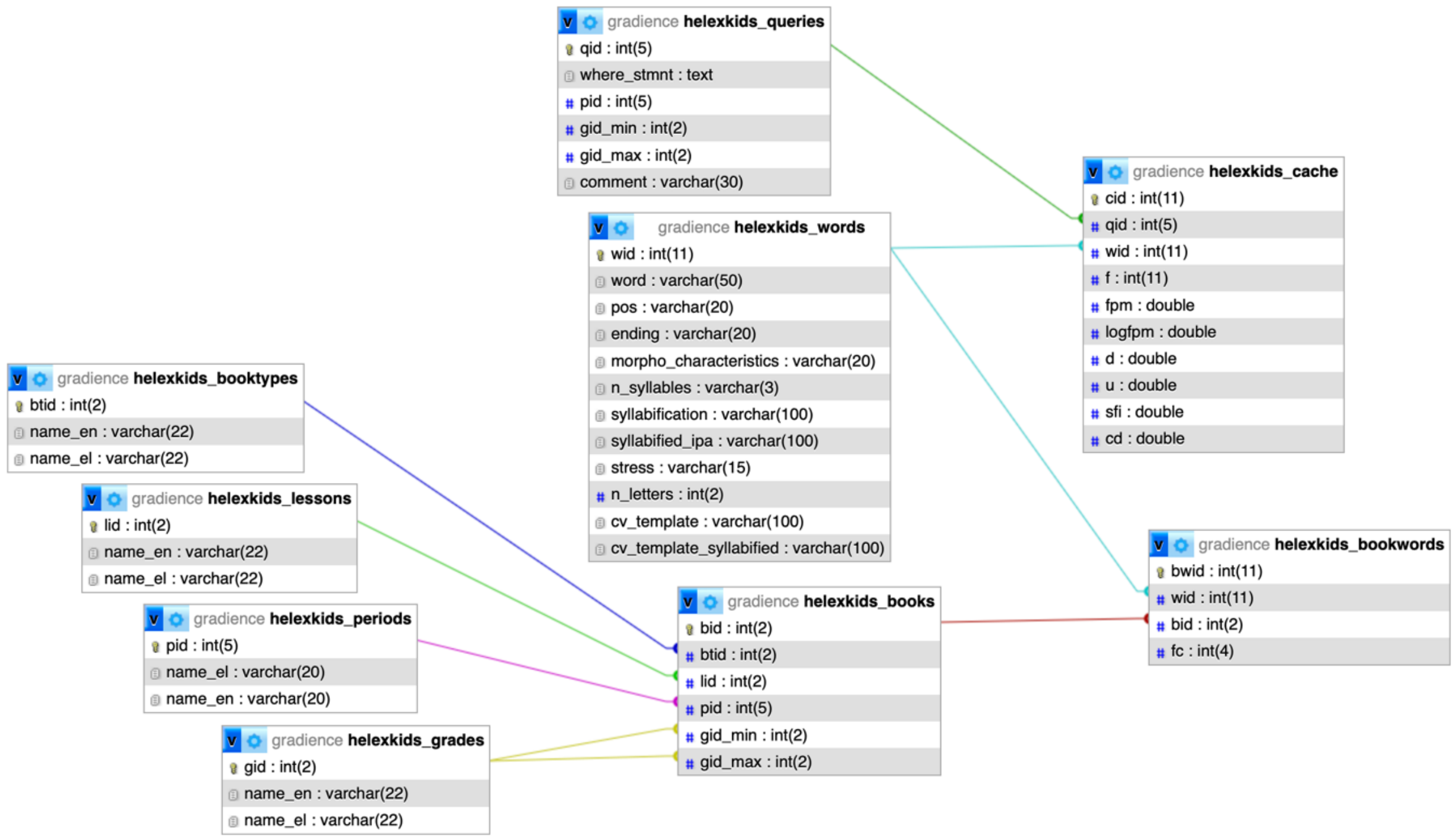

Figure A1. Relational schema of HelexKids 2.0, with lexical entries linked to textbooks, periods of textbook instruction, grade ranges, morphophonological annotations, and precomputed frequency measures.

Note. Connecting lines indicate primary and foreign key relationships between tables.

Core data tables:

helexkids_words (Central table): Contains 67,802 types with linguistic annotation. Each word is identified by a type ID (wid: int(11)) and includes: orthographic form (word: varchar(50)), part of speech (pos: varchar(20)), inflectional ending (ending: varchar(20)), morphological feature string (morpho_characteristics: varchar(20)), syllable count (n_syllables: varchar(3)),

syllabified form (syllabification: varchar(100)), IPA transcription with syllable boundaries (syllabified_ipa: varchar(100)), stress pattern (stress: varchar(15)), letter count (n_letters: int(2)), consonant-vowel template (cv_template: varchar(100)), and syllabified CV template (cv_template_syllabified: varchar(100)). This detailed annotation allows filtering by phonological, morphological, and orthographic criteria.

helexkids_cache: Stores pre-computed frequency and distributional statistics for word-query combinations. Each cache entry is identified by a cache ID (cid: int(11)) and links to a query ID (qid: int(5)) and word ID (wid: int(11)), containing: raw frequency count (f: int(11)), frequency per million words (fpm: double), log-transformed frequency per million (logfpm: double), dispersion index measuring spread across texts (d: double), usage coefficient (u: double), standard frequency index (sfi: double), and contextual diversity measuring the number of different contexts (cd: double). Pre-computing these metrics is necessary to improve query performance for large-scale frequency analyses. Periodic updates are performed by the database administrator.

helexkids_bookwords (Junction table): Links individual words to specific textbooks. Each entry is identified by a bookword ID (bwid: int(11)) and connects a word ID (wid: int(11)) to a book ID (bid: int(2)), storing the frequency count (fc: int(4)) of that word in that book. This table enables both aggregated database-level frequency calculations and book-specific vocabulary analyses.

Metadata and classification tables:

helexkids_books: Bibliographic metadata for 76 textbooks and workbooks in the database. Each book is identified by a book ID (bid: int(2)) and is classified by: book type ID (btid:

int(2), linking to helexkids_booktypes), lesson ID (lid: int(2), linking to helexkids_lessons), period ID (pid: int(5), linking to helexkids_periods), and grade range (gid_min, gid_max: int(2), linking to helexkids_grades). This multi-dimensional classification allows for sophisticated filtering by content type.

helexkids_booktypes: Lookup table for book type categories (e.g., language and arts textbooks, readers, workbooks) with book type ID (btid: int(2)) and bilingual labels (name_en, name_el: varchar(22)).

helexkids_lessons: Lookup table for curriculum units or lesson types, identified by lesson ID (lid: int(2)) with bilingual labels (name_en, name_el: varchar(22)). This structure enables filtering by pedagogical content area.

helexkids_periods: Defines the instructional period of the stored textbooks, identified by period ID (pid: int(5)) with bilingual labels (name_el, name_en: varchar(20)).

helexkids_grades: Grade level classifications corresponding to the Greek educational system, identified by grade ID (gid: int(2)) with bilingual labels (name_en, name_el: varchar(22)).

Query management and storage:

helexkids_queries: Stores parameterized query templates for common data extraction patterns. Each query is identified by a query ID (qid: int(5)).

Appendix B. Extended descriptive statistics

Table B1

Frequency distribution of PoS categories across types and tokens in HelexKids 2.0

PoS	Types (*N*)	Types (%)	Tokens (*N*)	Tokens (%)
NOUN	21,606	31.87	329,644	23.90
VERB	20,575	30.35	211,743	15.35
PRN/ART	11	0.02	185,380	13.44
CNJ	56	0.08	80,747	5.85
ADJ	10,452	15.42	64,543	4.68
PREP	48	0.07	52,435	3.80
ART	3	0.00	52,102	3.78
PRN/ADJ	218	0.32	40,634	2.95
PREP+ART	10	0.01	39,272	2.85
ADJ/NOUN	2,696	3.98	36,088	2.62
PNOUN	4,869	7.18	34,772	2.52
CNJ/PTCL	3	0.00	32,283	2.34
ADV	357	0.53	30,156	2.19
PRN	48	0.07	27,703	2.01
PREP/PRN	1	0.00	22,780	1.65
PRN/CNJ	13	0.02	22,542	1.63
ADJ/ADV	1,103	1.63	20,875	1.51
NUM/ART/PRN	12	0.02	19,843	1.44
PTCL	16	0.02	17,780	1.29

NUM	281	0.41	10,840	0.79
ADV/CNJ	10	0.01	8,036	0.58
OTHER	1,911	2.82	6,725	0.49
PCP/ADJ	2,120	3.13	6,216	0.45
VERB/NOUN	199	0.29	4,394	0.32
GER	666	0.98	3,826	0.28
ADJ/ADV/NOUN	95	0.14	3,753	0.27
CNJ/PREP	5	0.01	3,342	0.24
NOUN/PNOUN	130	0.19	1,577	0.11
ADV/DM	3	0.00	1,537	0.11
PREP/ADV	3	0.00	1,306	0.09
ADJ/PNOUN	65	0.10	1,074	0.08
ADV/PTCL/EXCL	3	0.00	957	0.07
PRN/ADJ/ADV	2	0.00	839	0.06
EXCL/DM	64	0.09	759	0.06
PREP/ADV/CNJ	2	0.00	656	0.05
CNJ/DM	1	0.00	606	0.04
ADV/NOUN	13	0.02	454	0.03
ADV/EXCL	4	0.01	391	0.03
PCP/ADJ/NOUN	57	0.08	228	0.02
ADV/PTCL	2	0.00	115	0.01
ADJ/ADV/CNJ	1	0.00	108	0.01
PCP/ADJ/ADV	15	0.02	85	0.01
NOUN/EXCL/DM	5	0.01	72	0.01

NUM/NOUN	5	0.01	60	0.00
ADJ/NOUN/PNOUN	5	0.01	53	0.00
NUM/ADV	2	0.00	41	0.00
NOUN/GER	1	0.00	26	0.00
ADJ/ADV/PREP	2	0.00	23	0.00
VERB/EXCL/DM	2	0.00	22	0.00
PRN/ADJ/NOUN	2	0.00	20	0.00
NUM/PNOUN	6	0.01	17	0.00
EXCL	3	0.00	10	0.00
VERB+PRN	7	0.01	9	0.00
ADJ/VERB	4	0.01	7	0.00
NOUN/EXCL	1	0.00	5	0.00
NOUN/PRN	2	0.00	3	0.00
ADJ/ADV/VERB	2	0.00	2	0.00
VERB/EXCL	1	0.00	2	0.00
ADJ/ADV/PNOUN	1	0.00	1	0.00
ADV/PRN	1	0.00	1	0.00
PNOUN/OTHER	1	0.00	1	0.00
**TOTAL**	**67,802**	**100**	**1,379,521**	**100**

Note. ADJ: Adjective, ADV: Adverb, ART: Article, CNJ: Conjunction, DM: Discourse Marker, EXCL: Exclamation, GER: Gerund, NOUN: Noun, NUM: Numeral, OTHER: Other, PCP: Participle, PNOUN: Proper Noun, PREP: Preposition, PRN: Pronoun, PTCL: Particle, VERB: Verb

Table B2

Stress pattern distribution by major PoS – types

PoS	APU	PU	U	Other
VERB	9,743 (47.35%)	8,181 (39.76%)	2,350 (11.42%)	301 (1.46%)
NOUN	5,815 (26.91%)	9,946 (46.03%)	4,887 (22.62%)	958 (4.43%)
ADJ	3,876 (37.08%)	1,072 (10.26%)	5,443 (52.08%)	61 (0.58%)
ADV	51 (14.29%)	190 (53.22%)	110 (30.81%)	6 (1.68%)

Note. "Other" includes unstressed or double-stressed words, and words with variant stress patterns.

Table B3

Stress pattern distribution by major PoS – tokens

PoS	APU	PU	U	Other
VERB	74,775 (35.31%)	113,811 (53.75%)	20,336 (9.60%)	2,821 (1.33%)
NOUN	79,680 (24.17%)	162,281 (49.23%)	80,395 (24.39%)	7,288 (2.21%)
ADJ	19,912 (30.85%)	9,045 (14.01%)	35,373 (54.81%)	213 (0.33%)
ADV	3,347 (11.10%)	17,520 (58.10%)	9,009 (29.87%)	280 (0.93%)

Note. "Other" includes unstressed or double-stressed words, and words with variant stress patterns.

Table B4

Cumulative stress pattern distribution in nouns – types

**Grade Range**	**APU**	**PU**	**U**	**Other**	**Types (*N*)**
1	709 (20.46%)	1,866 (53.85%)	787 (22.71%)	103 (2.97%)	3,465
1–2	1,110 (21.52%)	2,746 (53.24%)	1,148 (22.26%)	154 (2.99%)	5,158
1–3	2,091 (22.53%)	4,729 (50.95%)	2,140 (23.06%)	322 (3.47%)	9,282
1–4	3,619 (26.58%)	6,450 (47.37%)	3,079 (22.61%)	469 (3.44%)	13,617
1–5	5,095 (26.88%)	8,780 (46.33%)	4,286 (22.61%)	792 (4.18%)	18,953
1–6	5,815 (26.91%)	9,946 (46.03%)	4,887 (22.62%)	958 4.43	21,606

Note. "Other" includes unstressed or double-stressed words, and words with variant stress patterns.

Table B5

Cumulative stress pattern distribution in nouns – tokens

Grade Range	APU	PU	U	Other	Tokens (*N*)
1	3,306 (19.41%)	9,043 (53.10%)	4,390 (25.78%)	292 (1.71%)	17,031
1–2	6,775 (20.45%)	17,531 (52.91%)	8,177 (24.68%)	648 (1.96%)	33,131
1–3	17,445 (20.51%)	44,268 (52.04%)	21,543 (25.32%)	1,812 (2.13%)	85,068
1–4	32,686 (21.92%)	75,680 (50.76%)	37,394 (25.08%)	3,344 (2.24%)	149,104
1–5	57,581 (23.15%)	124,895 (50.21%)	60,626 (24.37%)	5,637 (2.27%)	248,739
1–6	79,680 (24.17%)	162,281 (49.23%)	80,395 (24.39%)	7,288 (2.21%)	329,644

Note. "Other" includes unstressed or double-stressed words, and words with variant stress patterns.

Table B6

Cumulative stress pattern distribution in verbs – types

**Grade Range**	**APU**	**PU**	**U**	**Other**	**Types (*N*)**
1	1,257 (40.73%)	1,446 (46.86%)	320 (10.37%)	63 (2.04%)	3,086
1–2	1,892 (41.70%)	2,079 (45.82%)	486 (10.71%)	80 (1.76%)	4,537
1–3	3,945 (45.32%)	3,671 (42.18%)	920 (10.57%)	168 (1.93%)	8,704
1–4	5,903 (45.55%)	5,370 (41.44%)	1,489 (11.49%)	196 (1.51%)	12,958
1–5	8,544 (47.19%)	7,265 (40.12%)	2,028 (11.20%)	269 (1.49%)	18,106
1–6	9,743 (47.35%)	8,181 (39.76%)	2,350 (11.42%)	301 (1.46%)	20,575

Note. "Other" includes unstressed or double-stressed words, and words with variant stress patterns.

Table B7

Cumulative stress pattern distribution in verbs – tokens

Grade Range	APU	PU	U	Other	Tokens (*N*)
1	3,751 (29.95%)	7,449 (59.48%)	983 (7.85%)	341 (2.72%)	12,524
1–2	7,329 (29.73%)	14,438 (58.57%)	2,348 (9.53%)	534 (2.17%)	24,649
1–3	21,175 (34.59%)	33,638 (54.94%)	5,261 (8.59%)	1,148 (1.88%)	61,222
1–4	36,929 (35.08%)	57,300 (54.42%)	9,506 (9.03%)	1,550 (1.47%)	105,285
1–5	59,305 (35.55%)	89,636 (53.73%)	15,567 (9.33%)	2,324 (1.39%)	166,832
1–6	74,775 (35.31%)	113,811 (53.75%)	20,336 (9.60%)	2,821 (1.33%)	211,743

Note. "Other" includes unstressed or double-stressed words, and words with variant stress patterns.

Table B8

Rank-ordered distribution of the top 10 word-level syllable templates by grade level, showing type counts and percentage of types

Grade	Template	Types (*N*)	Types (%)	Grade Types (*N*)	Rank
1	CV.ˈCV.CV.	441	4.86	9,077	1
1	ˈCV.CV.	354	3.9	9,077	2
1	ˈCV.CV.CV.	264	2.91	9,077	3
1	CV.CV.ˈCV.CV.	193	2.13	9,077	4
1	CV.ˈCV.	188	2.07	9,077	5
1	CV.ˈCV.CV.CV.	166	1.83	9,077	6
1	ˈCCV.CV.	166	1.83	9,077	7
1	ˈCV.CVC.	141	1.55	9,077	8
1	CCV.ˈCV.CV.	130	1.43	9,077	9
1	CV.CV.ˈCV.	129	1.42	9,077	10
2	CV.ˈCV.CV.	481	4.16	11,575	1
2	ˈCV.CV.	416	3.59	11,575	2
2	ˈCV.CV.CV.	309	2.67	11,575	3
2	CV.CV.ˈCV.CV.	223	1.93	11,575	4
2	CV.ˈCV.	219	1.89	11,575	5
2	CV.ˈCV.CV.CV.	202	1.75	11,575	6
2	ˈCCV.CV.	201	1.74	11,575	7
2	ˈCV.CVC.	185	1.6	11,575	8
2	CV.CV.ˈCV.	164	1.42	11,575	9

2	CCV.ˈCV.CV.	150	1.3	11,575	10
3	CV.ˈCV.CV.	666	3.06	21,759	1
3	ˈCV.CV.	569	2.62	21,759	2
3	ˈCV.CV.CV.	518	2.38	21,759	3
3	CV.ˈCV.CV.CV.	370	1.7	21,759	4
3	CV.CV.ˈCV.CV.	351	1.61	21,759	5
3	ˈCV.CVC.	337	1.55	21,759	6
3	ˈCCV.CV.	291	1.34	21,759	7
3	CV.ˈCV.	252	1.16	21,759	8
3	CV.CV.ˈCV.	245	1.13	21,759	9
3	CV.ˈCV.CVC.	232	1.07	21,759	10
4	CV.ˈCV.CV.	846	2.54	33,322	1
4	ˈCV.CV.CV.	679	2.04	33,322	2
4	ˈCV.CV.	665	2	33,322	3
4	CV.ˈCV.CV.CV.	543	1.63	33,322	4
4	CV.CV.ˈCV.CV.	469	1.41	33,322	5
4	ˈCV.CVC.	379	1.14	33,322	6
4	ˈCCV.CV.	364	1.09	33,322	7
4	CV.CV.ˈCV.	355	1.07	33,322	8
4	V.ˈCV.CV.CV.	343	1.03	33,322	9
4	CV.ˈCV.	334	1	33,322	10
5	CV.ˈCV.CV.	1011	2.28	44,383	1
5	ˈCV.CV.CV.	821	1.85	44,383	2
5	ˈCV.CV.	773	1.74	44,383	3

5	CV.ˈCV.CV.CV.	704	1.59	44,383	4
5	CV.CV.ˈCV.CV.	581	1.31	44,383	5
5	V.ˈCV.CV.CV.	447	1.01	44,383	6
5	ˈCCV.CV.	446	1	44,383	7
5	ˈCCV.CV.CV.	442	1	44,383	8
5	ˈCV.CVC.	435	0.98	44,383	9
5	CV.CV.ˈCV.	432	0.97	44,383	10
6	CV.ˈCV.CV.	1069	2.25	47,511	1
6	ˈCV.CV.CV.	848	1.78	47,511	2
6	ˈCV.CV.	784	1.65	47,511	3
6	CV.ˈCV.CV.CV.	745	1.57	47,511	4
6	CV.CV.ˈCV.CV.	637	1.34	47,511	5
6	ˈCV.CVC.	491	1.03	47,511	6
6	CV.CV.ˈCV.	481	1.01	47,511	7
6	V.ˈCV.CV.CV.	474	1	47,511	8
6	CV.ˈCV.	457	0.96	47,511	9
6	ˈCCV.CV.	454	0.96	47,511	10

Table B9

Rank-ordered distribution of the top 10 word-level syllable templates by grade level, showing token counts, corresponding types, percentage of tokens, and total grade token counts

Grade	Template	Tokens (*N*)	Types (*N*)	Tokens (%)	Grade Tokens (*N*)	Rank
1	CV.	16,015	34	22.77	70,345	1
1	ˈCV.CV.	3,354	354	4.77	70,345	2
1	ˈV.CV.	2,847	62	4.05	70,345	3
1	V.	2,829	7	4.02	70,345	4
1	CVC.	2,503	14	3.56	70,345	5
1	CV.ˈCV.	1,935	188	2.75	70,345	6
1	CV.ˈCV.CV.	1,741	441	2.47	70,345	7
1	CVN.	1,611	13	2.29	70,345	8
1	CCV.	1,310	6	1.86	70,345	9
1	V.ˈCV.	1,205	22	1.71	70,345	10
2	CV.	21,704	34	22.64	95,886	1
2	ˈCV.CV.	4,291	416	4.48	95,886	2
2	ˈV.CV.	3,731	64	3.89	95,886	3
2	CVC.	3,286	12	3.43	95,886	4
2	V.	3,092	6	3.22	95,886	5
2	CV.ˈCV.	2,810	219	2.93	95,886	6
2	CVN.	2,092	13	2.18	95,886	7
2	CV.ˈCV.CV.	1,975	481	2.06	95,886	8

2	CCV.	1,725	8	1.8	95,886	9
2	ˈCV.CV.CV.	1,715	309	1.79	95,886	10
3	CV.	49,998	38	21.73	230,041	1
3	V.	9,511	8	4.13	230,041	2
3	CVC.	9,370	21	4.07	230,041	3
3	ˈCV.CV.	9,333	569	4.06	230,041	4
3	ˈV.CV.	7,696	89	3.35	230,041	5
3	CVN.	6,273	15	2.73	230,041	6
3	CV.ˈCV.	5,142	252	2.24	230,041	7
3	V.ˈCV.	4,305	34	1.87	230,041	8
3	ˈCV.CV.CV.	4,115	518	1.79	230,041	9
3	CV.ˈCV.CV.	3,880	666	1.69	230,041	10
4	CV.	68,716	40	20.32	338,202	1
4	CVC.	13,598	22	4.02	338,202	2
4	V.	12,795	8	3.78	338,202	3
4	ˈCV.CV.	12,551	665	3.71	338,202	4
4	ˈV.CV.	11,535	101	3.41	338,202	5
4	CVN.	9,023	14	2.67	338,202	6
4	CV.ˈCV.	7,531	334	2.23	338,202	7
4	V.ˈCV.	6,018	45	1.78	338,202	8
4	ˈCV.CV.CV.	5,711	679	1.69	338,202	9
4	CV.ˈCV.CV.	5,361	846	1.59	338,202	10
5	CV.	91,176	45	19.67	463,502	1
5	CVC.	18,952	30	4.09	463,502	2

5	V.	16,868	9	3.64	463,502	3
5	ˈCV.CV.	16,148	773	3.48	463,502	4
5	ˈV.CV.	15,974	120	3.45	463,502	5
5	CVN.	13,969	19	3.01	463,502	6
5	CV.ˈCV.	9,450	427	2.04	463,502	7
5	V.ˈCV.	8,374	64	1.81	463,502	8
5	ˈCV.CV.CV.	7,581	821	1.64	463,502	9
5	ˈCCV.CV.	6,788	446	1.46	463,502	10
6	CV.	105,510	50	19.8	532,749	1
6	CVC.	20,825	36	3.91	532,749	2
6	V.	20,143	9	3.78	532,749	3
6	ˈV.CV.	18,706	121	3.51	532,749	4
6	ˈCV.CV.	17,995	784	3.38	532,749	5
6	CVN.	16,406	22	3.08	532,749	6
6	CV.ˈCV.	11,040	457	2.07	532,749	7
6	V.ˈCV.	9,633	65	1.81	532,749	8
6	ˈCV.CV.CV.	8,084	848	1.52	532,749	9
6	CCV.	7,898	12	1.48	532,749	10
